# High-throughput DNA analysis shows the importance of methylation in the control of immune inflammatory gene transcription in chronic periodontitis

**DOI:** 10.1186/1868-7083-6-15

**Published:** 2014-08-12

**Authors:** Ana Paula De Souza, Aline Cristiane Planello, Marcelo Rocha Marques, Daniel Diniz De Carvalho, Sergio Roberto Peres Line

**Affiliations:** 1 Department of Morphology, School of Dentistry of Piracicaba, FOP-UNICAMP, University of Campinas, Av. Limeira 901, Piracicaba, SP 13414-018, Brazil; 2 Ontario Cancer Institute, Princess Margaret Hospital, University Health Network, Toronto, ON M5G 2M9, Canada

**Keywords:** Chronic periodontitis, DNA methylation, Epigenetics, Inflammation, Transcription

## Abstract

**Background:**

Chronic periodontitis represents a complex disease that is hard to control and is not completely understood. Evidence from past studies suggests that there is a key role for DNA methylation in the pathogenesis of periodontitis. However, all reports have applied technologies that investigate genes in a low throughput. In order to advance in the knowledge of the disease, we analyzed DNA methylation variations associated with gene transcription using a high-throughput assay. Infinium® HumanMethylation450 (Illumina) was performed on gingival samples from 12 periodontitis cases and 11 age-matched healthy individuals. Methylation data of 1,284 immune-related genes and 1,038 cell cycle-related genes from Gene Ontology (GO) and 575 genes from a dataset of stably expressed genes (genes with consistent expression in different physiological states and tissues) were extracted from a microarray dataset and analyzed using bioinformatics tools. DNA methylation variations ranging from −2,000 to +2,000 bp from the transcription start site (TSS) were analyzed, and the results were tested against a differential expression microarray dataset between healthy and periodontitis gingival tissues. Differences were evaluated using tests from the R Statistical Project.

**Results:**

The comparison of probes between periodontitis and normal gingival tissues showed that the mean methylation scores and the frequency of methylated probes were significantly lower in genes related to the immune process. In the immune group, these parameters were negatively correlated with gene expression (Mann-Whitney test, *p* < 2.2e − 16).

**Conclusions:**

Our results show that variations in DNA methylation between healthy and periodontitis cases are higher in genes related to the immune-inflammatory process. Thus, DNA methylation must be modulating chromatin regions and, consequently, modulating the mRNA transcription of immune-inflammatory genes related with periodontitis, impacting the prognosis of disease.

## Background

Chronic periodontitis (or chronic periodontal disease) represents inflammation of the periodontium, the connective tissue that attaches the surface of root teeth to the alveolar bone. As a major health problem, periodontitis is widely regarded as the second most common disease worldwide, affecting 30% to 50% of the adult population in occidental countries [1]. The most common consequence of periodontitis is the loss of one or more teeth in severe cases. Furthermore, periodontitis has been identified as a risk factor for lethal diseases such as endocarditis, stroke, myocardial infarction, and atherosclerosis [2-6].

It is well known that periodontitis is associated with the presence of bacteria biofilm on the teeth and oral mucosa surfaces [6]. It is also well known which environmental factors, social habits, some drugs, systemic health, and smoking influence the prognosis of periodontitis. Likewise, hundreds of studies published in the last decade have reported that genetic background is a key modulator of the host's response to the infection [7-11].

Recently, a growing number of articles have dedicated special attention to epigenetic changes in DNA, stressing the importance of the ‘epigenetic phenotype’ on diverse diseases. Methylation, the addition of a methyl group to cytosine at CpG dinucleotides, is the most common epigenetic modification in DNA. CpG sites are not randomly distributed in the human genome; they are enriched in regions defined as CpG islands (CGI) and represent regions of at least 200 bp containing a proportion of guanine and cytosine greater than 50% and are observed at an expected CpG ratio greater than 0.6 [12]. CGIs, which are concentrated in the promoters of genes, are the first candidates to be investigated in gene expression-methylation studies [13,14]. In addition to CGIs, it has been shown that methylation on sequences up to 2 kb distant from CGI (shore islands) and sequences up to 2 kb distant from shore (shelve islands) can influence gene expression [15,16].

Considering that DNA methylation is observed in gene promoters, around the transcription start site (TSS), gene bodies, and shore and shelve islands, and given that those regions can be functionally important [16,17], this study analyzed the variation of methylation between normal gingival and periodontitis tissues in a region spanning down 2,000 to up 2,000 bp from TSS in three groups of genes: genes involved with immune-inflammatory process, cell-cycle controlling genes, and stably expressed genes. The genes were analyzed using a dataset derived from microarray chips (Infinium®HumanMethylation 450 BeadChip from Illumina). Our results show that variations in DNA methylation between healthy and periodontitis tissues is higher in genes related to the immune-inflammatory process. Differences in DNA methylation variations between genes related to the immune-inflammatory process and the other groups of genes are concentrated in sequences near the TSS, and variations in DNA methylation in genes related to the immune-inflammatory process are correlated with variations in mRNA levels. To the best of our knowledge, this is the first high-throughput study showing consistent evidence of the key role of DNA methylation in the transcription level of immune-inflammatory genes involved in chronic periodontitis.

## Results

**Figure 1:**
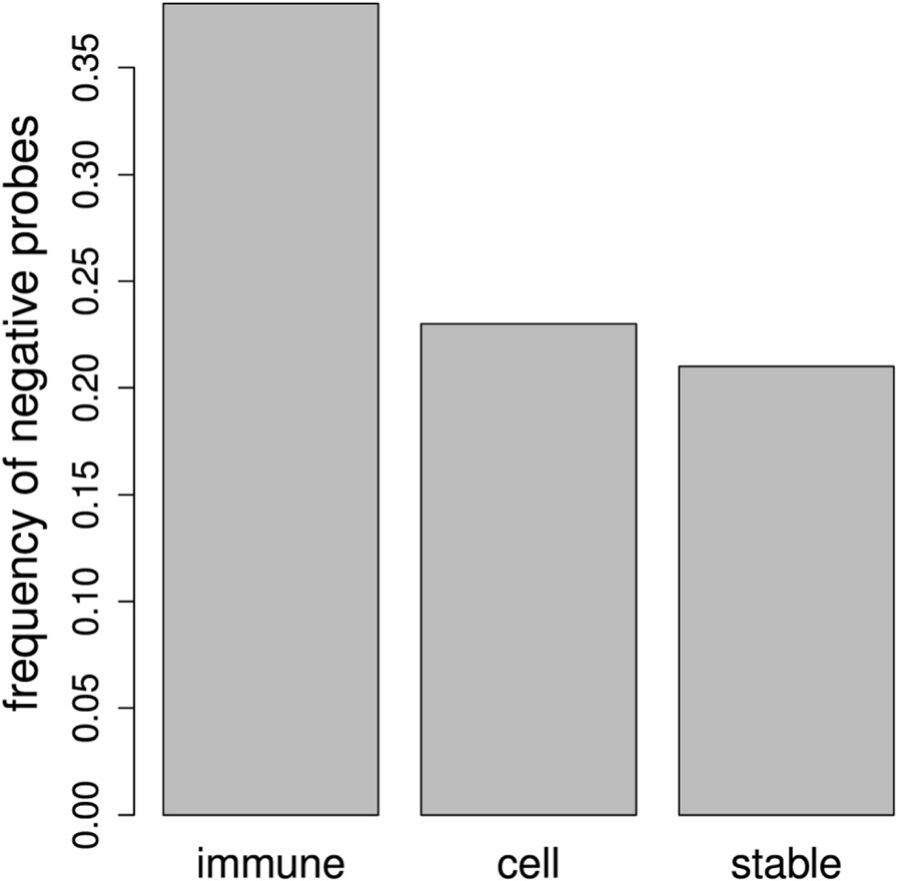
**Comparison of the variation in the methylation of probes in the region between +2,000 and −2,000.** Probes related to the immune-inflammatory genes (*n* = 5422), cell cycle (*n* = 4538), and stably expressed genes (*n* = 2349). Note that the frequency of probes with decreased methylation in periodontitis was higher in genes related to the immune system process (chi-squared test, *p* < 2.2e − 16).

A total of 59,999 probes were analyzed adding the three groups of genes, and 12,049 probes presented significant differences (*q* values < 0.05) when comparing samples from normal and periodontitis individuals. The comparison of the variation in the sign of methylation of the significant probes in sequences spanning from +2,000 to −2,000 among the three groups showed that *immune* group genes had significantly more negative probes (2,026 negative, 3,285 positive) than did the *cell cycle* gene (1,010 negative, 3,427 positive), and *stable* gene groups (485 negative, 1,816 positive) (chi-squared test, *p* < 2.2e − 16), showing that the frequency of probes with decreased methylation in periodontitis was higher in genes related to immune system process (Figure 1).

**Figure 2:**
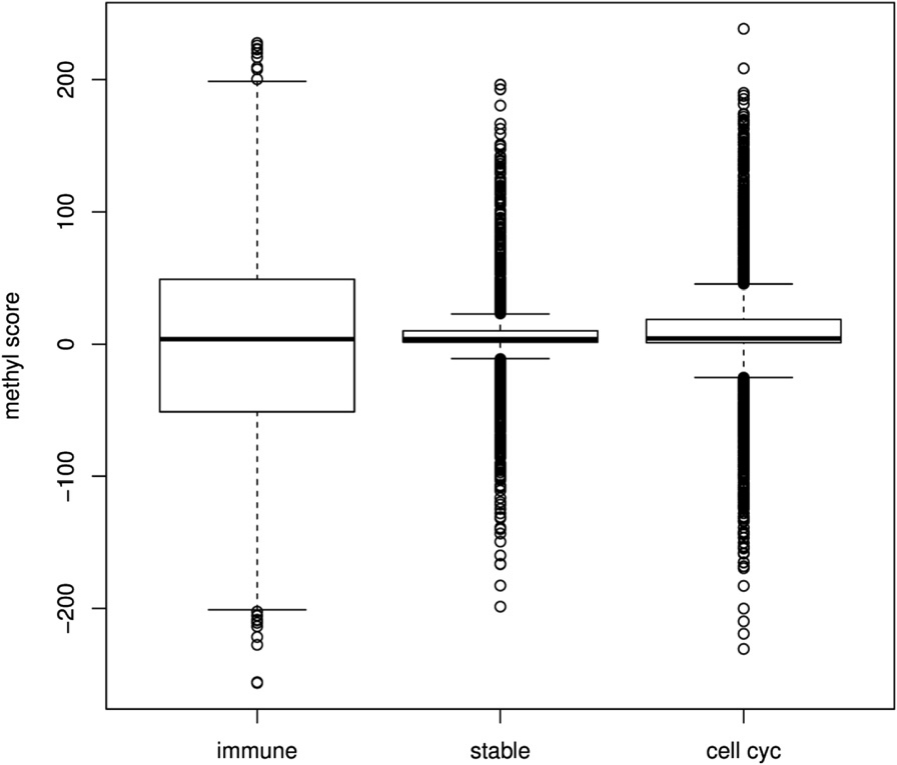
**Comparison of methyl scores in the region between +2,000 and −2,000.** Boxplot showing the median and interquartile range of methyl scores of probes related to the immune-inflammatory genes (*n* = 5,422), cell cycle (*n* = 4,538), and stably expressed genes (*n* = 2,349). Note that the variation of probes in the immune group, seen by the breadth of quartiles, is higher than in the stable and cell cycle groups (*p* < 2.2e − 16).

**Figure 3:**
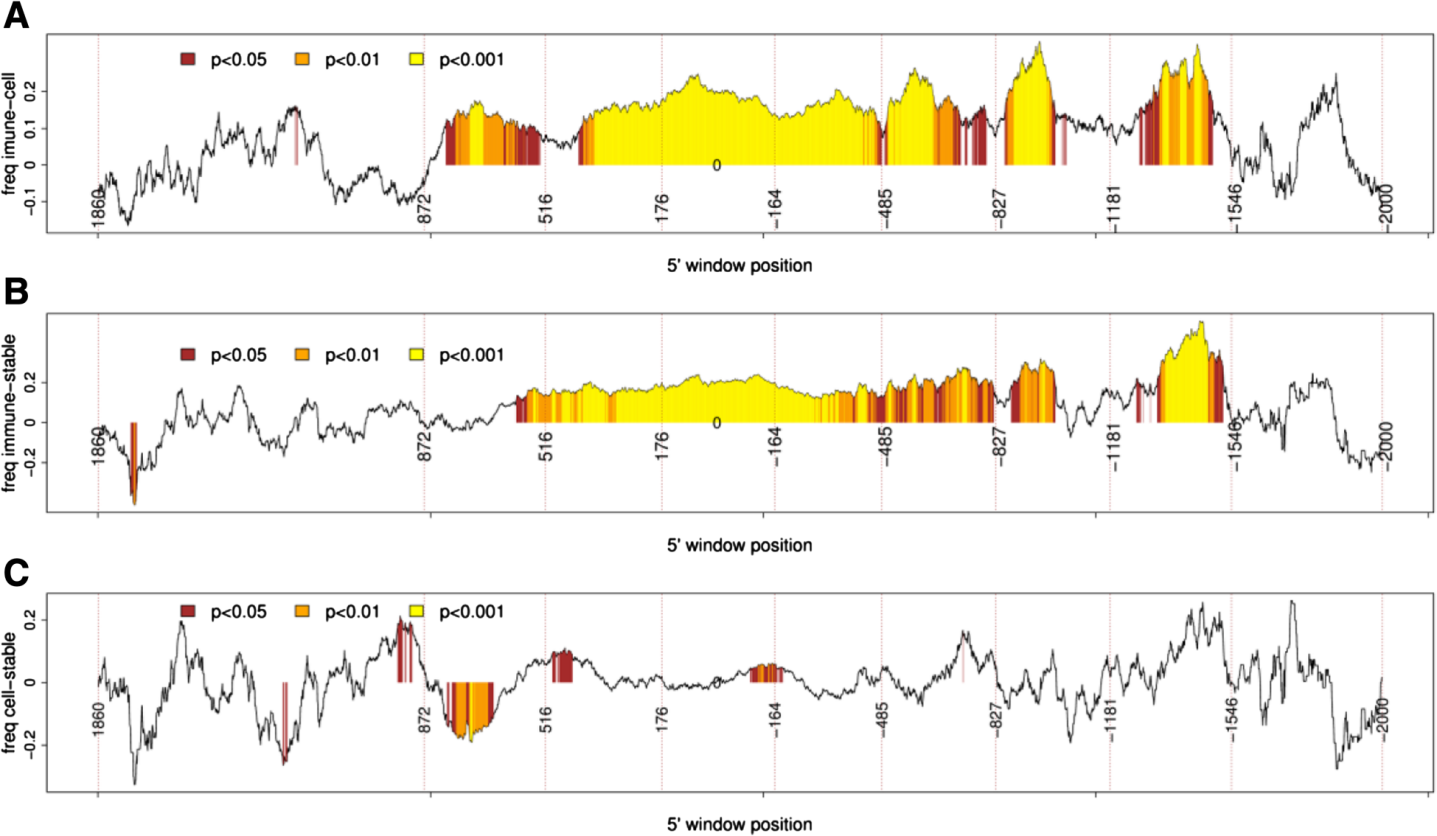
**Comparative analysis of variations in the frequency of negative probes in the groups.** The position of probes in the compared groups was matched using a sliding window of 140 bases (with a shift of one base) starting at +2,000 to +1,860 (within gene) and ending at a window containing sequences from −1,860 to −2,000 (promoter). Each point represents the difference between the frequency of the negative probes between the two groups. Significant differences (Mann-Whitney test) are shown in different colors. The 5′ position of the window is shown by vertical dotted lines. The larger regions with significant differences were observed when subtracting values: immune − cell cycle **(A)**, immune − stable **(B)**, and **(C)** cell cycle − stable. The *x*-axis positions larger than 0 represent gene bodies and less than 0 represent promoter regions.

**Figure 4:**
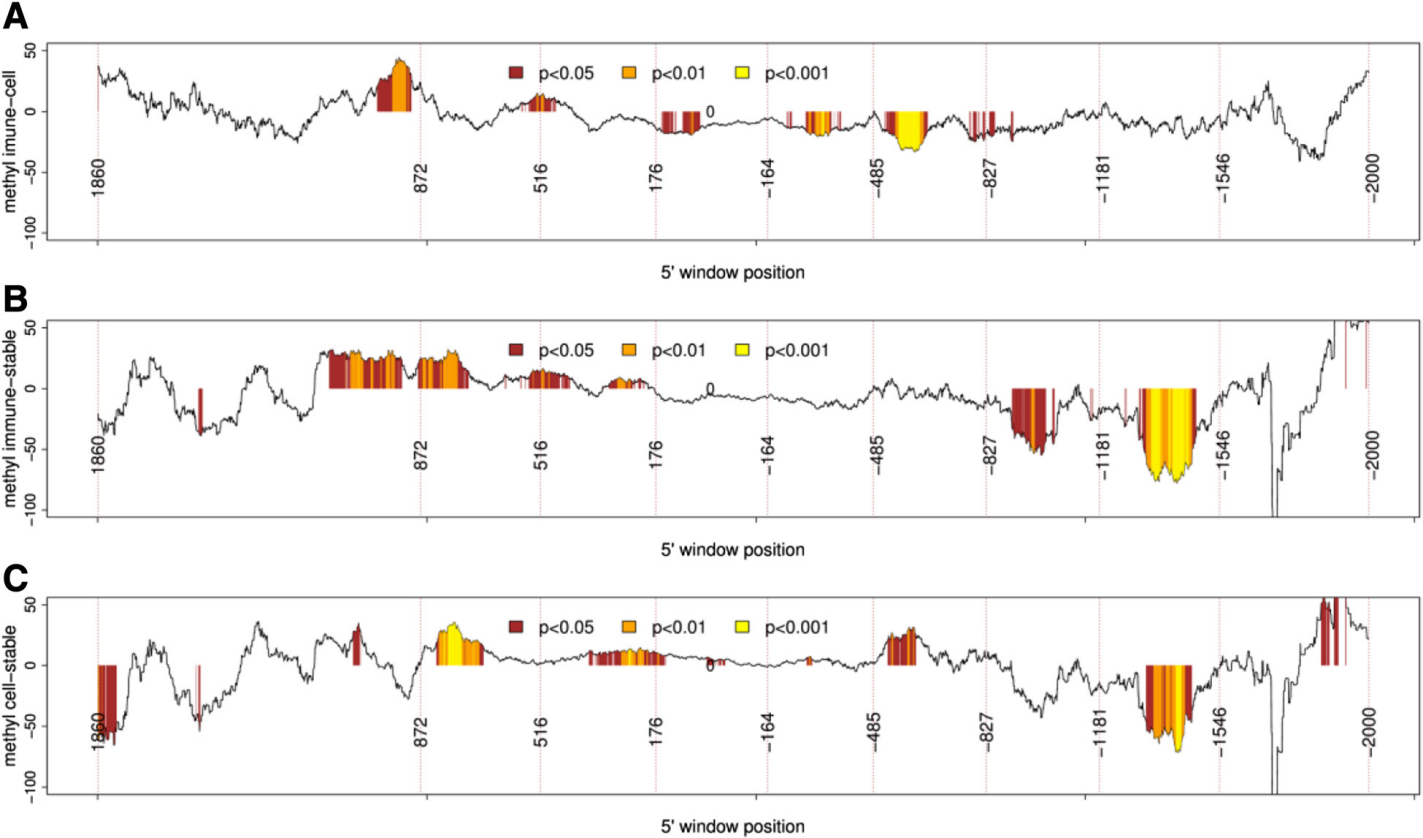
**Comparative analysis of variations in methyl scores in the immune, cell cycle, and stable groups.** The position of probes in the compared groups was matched using a sliding window of 140 bases (with a shift of one base) starting at +2,000 to +1,860 (within gene) and ending at a window containing sequences from −1,860 to −2,000 (promoter). Each point represents the difference between the methyl score between the two groups. Significant differences (Mann-Whitney test) are shown in different colors. The 5′ position of the window is shown by vertical dotted lines. The *x*-axis positions larger than 0 represent gene bodies and less than 0 represent promoter regions. **(A)** Methyl score immune − cell cycle, **(B)** methyl score immune − stable, and **(C)** methyl score cell cycle − stable.

Also, the immune group genes had a higher variation in the methyl score than the other two groups (*p* < 2.2e − 16, *F* test with confidence level = 0.95), indicating that the variations in methylation in genes from the immune group were significantly higher than in the other groups (Figure 2).We then sought to determine whether the variations of the methyl scores and the frequency of negative probes (the frequency of probes with decreased methylation in periodontitis) among the three groups of genes could be mapped to specific gene regions. For that, we subtracted the frequency of negative probes and the methyl scores between the two groups (immune − cell cycle, immune − stable, and immune − cell cycle). The positions of the probes in the two compared groups were matched using a sliding window of 140 bases (with a shift of one base). The first window comprised the region from +2,000 to +1,860 (within the gene) and the last window contained the sequences between −1,860 and −2,000 (promoter). The comparisons showed that genes related to the immune process were significantly less methylated (a higher frequency of negative probes) than the other two groups in the region spanning approximately +900 to −1,500 (Figures 3 and 4). Interestingly, the differences in the frequency of negative probes between the immune and cell cycle groups seem to be split in peaks and valleys with a period of approximately 350 base pairs, evidenced by the vertical dotted lines. The genes of the stable group were significantly more methylated (negative values) than those in the cell cycle group in the sequences ranging from approximately +870 to +600. The differences between methyl scores were more restricted than were the differences between the frequencies of the negative probes (Figures 3 and 4), indicating that the frequency of the probes with negative scores was a better discriminant parameter than methyl score.

**Figure 5:**
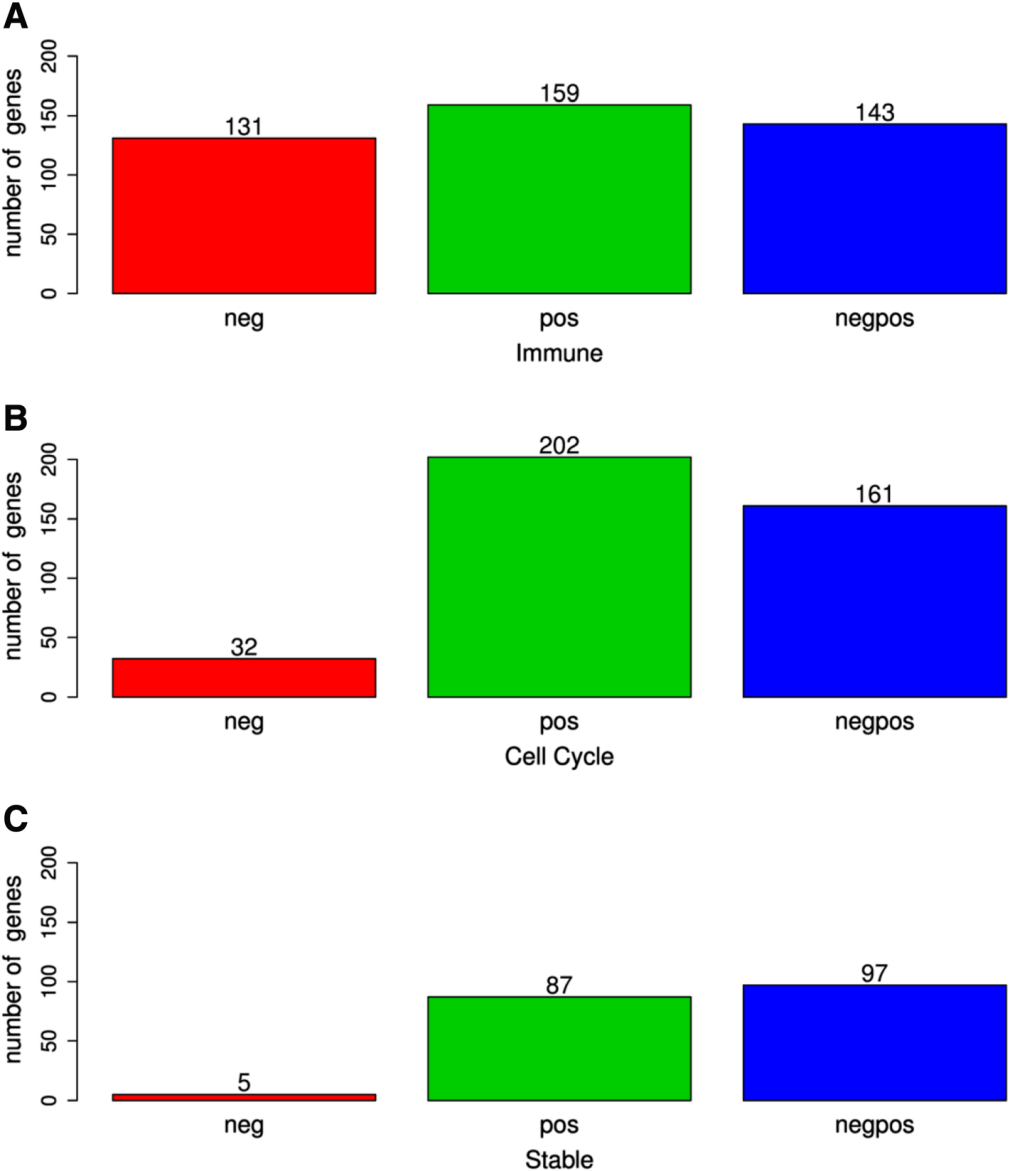
**Pattern of methyl score sign in the promoter region (0 to −1,700) of all three groups.** Genes with probes with only negative methyl scores (neg), only positive methyl scores (pos), and both positive and negative methyl scores (negpos). **(A)** Number of immune-inflammatory genes. **(B)** Number of cell cycle genes. **(C)** Number of stably expressed genes. Note that the frequency of genes with only negative methyl scores was higher in the immune group (30.9%) than in the cell cycle (8.9%) and stable (2.7%) groups, indicating a higher frequency of clustered demethylation of the genes related to the immune process during periodontitis (chi-squared test, *p* < 2.2e − 16).

We also analyzed the pattern of the methylation of probes in the region located between 0 and −1,700 (promoter). To accomplish that, we selected genes with more than two probes with significant methyl scores (*q* value ≤ 0.05). Genes were divided into three categories according to the homogeneity of the sign of the probes: ‘neg’, all the probes in the promoter region were negative (decreased methylation in periodontitis); ‘pos’, all the probes in the promoter region were positive (increased methylation in periodontitis); and ‘negpos’, with both positive and negative probes. Interestingly, most genes showed homogeneity in the sign of probes in the promoter region, where all the probes were positive (increase in methylation or pos) or all probes were negative (decrease in methylation or neg). This pattern will be referred to, hereafter, as a cluster phenomenon. The frequency of the cluster phenomenon ([neg + pos]/[neg + pos + negpos]) in the immune, cell cycle, and stable groups was 66.97% (290/433), 59.24% (234/395), and 48.68% (92/189), respectively. These results indicate that this may be a common phenomenon in human promoters. The proportion of genes with only negative probes was higher in the immune group (30.25%, 131/433) than in the cell cycle (8.10%, 32/395) and stable (2.64%, 5/189) groups (Figure 5A). In order to check if the frequency of genes with only negative probes was higher in the immune group, we compared the methyl scores of the cell cycle and stable groups and of the pos and negpos categories. A comparison of frequencies with Pearson's chi-squared analysis showed a highly significant increase of negative probes in the immune group (*p* < 2.2e − 16). Also interesting is the observation of the cluster phenomenon in the promoter regions of the cell cycle and stable groups. In most genes (58.9% for cell cycle and 52.0% for stable), all significant probes were positive (Figure 5A). This may be related to the fact that inflammatory cells, which predominate in periodontitis sites, are non-proliferative, with a predominance of destructive, over-reparative processes in the disease site. Therefore, it is possible that in inflammatory cells, increased DNA methylation in genes of the cell cycle and *stable expression* groups is associated with a tendency of transcriptional downregulation.

**Figure 6:**
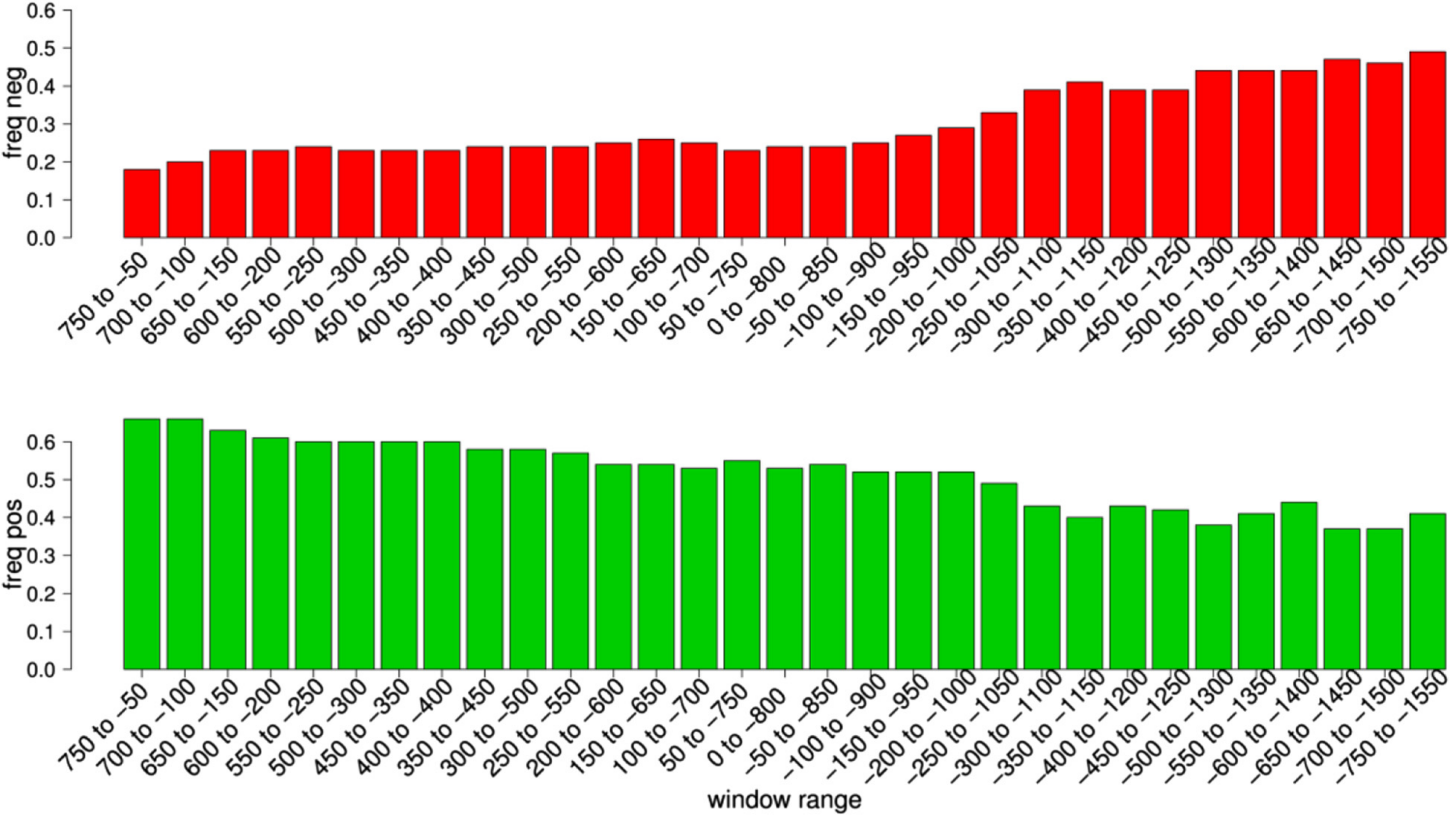
**Cluster phenomenon analysis in the promoter region of genes from the immune group.** Each bar shows the frequency of genes where all of the probes were negative (only windows with more than three probes were included in the analysis). The position of the probes in the compared groups was matched using a sliding window of 800 bases (with a shift of 50 bases). The upper chart shows the frequency of genes where all of the probes were negative. The lower chart shows the frequency of genes where all of the probes were positive. Note that the frequency of genes where all of the probes were negative (decrease in methylation) was highest in the regions located between −600 and −1,550, while the frequency of genes where all of the probes were positive was highest in the regions located between −750 and −550.

In order to better characterize the cluster phenomenon, we have scanned the sequences of genes from the immune group from +750 to −1,500 using a sliding window of 800 bases (with a shift of 50 bases). The frequency of genes where all the probes were negative (decreased methylation in chronic periodontitits) was maximal in the regions located between −600 and −1,550 (Figure 6), while the frequency of genes where all the probes were positive was maximal in the regions located between +750 and −550 (Figure 6, Additional file 1: Figure S1, Additional file 2: Figure S2). These results suggest that cluster CpG methylation can have relevant biological significance in areas more distant from the TSS.

**Figure 7:**
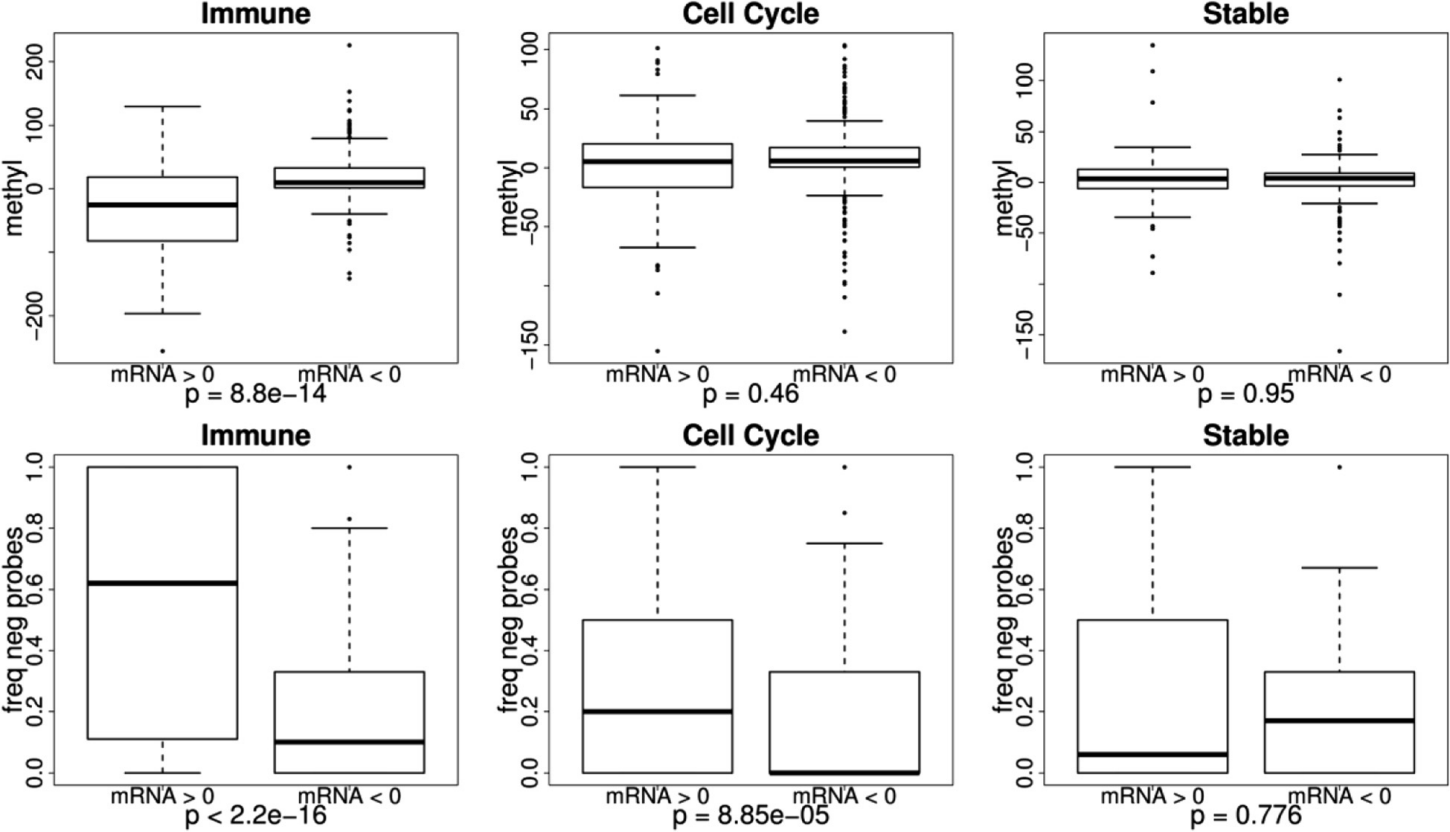
**Methylation variation and mRNA expression.** Variations in methyl score (methyl) and the frequency of negative probes (freqneg probes) in genes with increased (mRNA > 0) and genes with decreased (mRNA < 0) expression in periodontitis when compared to normal gingival tissues. The analysis included probes in the region spanning from +2,000 to −2,000.

There was a significant association between variations in gene expression and methylation in the immune group, where genes with increased expression in periodontitis tended to have lower methyl scores (*p* = 8.8e − 14; upper line of Figure 7) and higher frequency of negative probes (*p* < 2.2e − 16; lower line of Figure 7). Both experiments indicate that there is an inverse association between the variations in methylation and mRNA levels. This tendency was also observed for the frequency of negative probes in the group of cell cycle genes (*p* = 8.85e − 5), while no significant differences were found in the group of stable genes.

In order to verify whether the association between variations in mRNA levels and methylation could be mapped to specific gene regions, we performed correlation analysis between variations in methylation and mRNA levels using a sliding window of 140 bp (with a shift of one base). Since both the frequencies of negative probes and methyl score are associated with variations in mRNA levels, we tested these parameters either in isolation or by assessing the interaction between them (i.e., the frequency of negative probes × methyl score). Higher correlation values and more significant correlations (smaller *p* values) were found in the interaction of the two factors, indicating that both parameters are relevant for gene expression. The correlation analysis between variations in mRNA levels and methylation in the immune group (Figure 7) exhibited a large region spanning approximately +900 to −1,000, where most windows showed a significant negative correlation between variations in methylation and mRNA levels. In this region, the highest correlation was −0.50 (*p* = 3.3e − 10), which was found in the window −416 to −276 with 139 genes. This region contained most of the significant areas shown in Figures 3A,B and 4A,B. Smaller regions with negative correlations were also observed in the cell cycle group (Figure 7).

## Discussion

Using the microarray dataset and bioinformatics tools, we performed a high-throughput analysis of DNA methylation variation across three different classes of genes from GO annotation - immune–inflammatory, cell cycle, and stable genes - and we correlated the findings with the gene expression of normal gingival tissue and gingival tissue from chronic periodontitis-affected individuals. We found important evidence of the role of DNA methylation on the transcriptional regulation of immune genes during chronic periodontitis that was not as evident for the cell cycle and stable genes. The results clearly show that the variations in methylation status between healthy and periodontitis cases were more significant in genes related to the immune system process, which showed a higher tendency for DNA unmethylation than did the genes of the other two groups. We found an inverse correlation between variations in methylation and the mRNA levels of the immune genes during periodontitis, which occurred in a similar magnitude to the ones obtained by Su and collaborators [18].

The assays in this study were performed using entire gingival tissue, containing epithelial and connective tissue cells, including blood cells. Thus, the observed variations in the methylation between normal and diseased tissues likely occurred as a result of two distinct phenomena: (1) Changes occurred in cell populations between healthy and inflamed tissues. While in normal tissues there is a predominance of fibroblasts and epithelial cells, the major cell types in inflamed tissues are leukocytes and endothelial cells: (2) Changes occurred in the methylation pattern caused by the activation of resident and incoming fibroblasts, macrophages, and epithelial cells. In the resident cells, the set of expressed genes can be reorganized under influence of inflammatory mediators, which requires epigenetic changes. We have detected a significant upregulation in the mRNA levels of enzymes that modify chromatin, DNMT1 and TET1, in the periodontitis tissue samples (data not shown). The increased expression of TET1 is especially interesting, as this enzyme may promote passive demethylation [16]. The unmethylated state of CpG islands is presumably archived by the activity of the TET1 enzyme, which converts any 5-methycytosine into 5-hydroxymethylcytosine [16,19,20]. Following this, the nuclear DNA repair machinery excises 5-hydroxymethylcytosine to replace a cytosine nucleotide in the DNA strand [21], furthering demethylation events. However, these data need further confirmation by other methods.

Comparing periodontitis versus normal gingival tissue, we detected significant differences in methylation variation in pro-inflammatory genes, including a wide range of cytokines, cytokine receptors, receptor antagonists, growth factors, transcription factors, and cell membrane proteins, among other genes implicated in the immune response. We also detected methylation variations in the key genes of T cell differentiation, such as GATA3, IL4, IL5, IL12, and IL17A (see Additional file 3: Table S1). The establishment of the T cell subset type may be important for determining disease prognosis. The cell-mediated immunity profile (TH1 cell response) has pro-inflammatory characteristics, having IFN-γ described as the key signature cytokine of the TH1-type response [22]. Already, the antibody-mediated immunity profile (TH2 cell response) comes up as an anti-inflammatory phenotype [23]. The expression of the T-bet and GATA3 gene is an important step to drive the differentiation of naive T cells [23]. The concomitant expression of T-bet and GATA3 induces T cell division and the acquisition of a mixed Th1 and Th2 lineage. Following that, with further mitosis, the responsiveness to cytokines and growth factor signals will select the predominant Th profile, which may be a deciding factor in determining whether periodontal therapy is successful.

The epigenetic control on naive T cell differentiation has been demonstrated. Bird and collaborators [24] had shown that helper T cell differentiation is controlled by the cell cycle, and other authors have reasoned that the cell cycle and DNA synthesis, in particular, would provide a window of opportunity to remodel genes from an inactive to an active state in naive T cells [25]. In fact, the role of epigenetic effects in silencing or activating genes that control the immune response has been known for decades [23,26], and authors have been emphatic about the importance of DNA and chromatin-modifying factors in controlling the expression of immune-inflammatory genes [23,27,28]. Our results reinforce the importance of epigenetic control and show, for the first time in a global context, the impact of DNA methylation on the transcription of immune-inflammatory genes by resident and immune cells of chronic periodontitis sites.

We also mapped regions that probably modulate gene function. Working with a sliding window of 140 bp, we observed that many regions around the promoter, which neighbor the TSS, and inside the gene body show frequencies of negative probes and values of methyl scores that are significantly different for the immune-inflammatory genes. A recently published review [16] discussed the functions of DNA methylation in enriched CGI promoters both around the TSS site and inside the gene body. The author concluded that the relationship between DNA methylation and gene control can vary across different genes, since methylation in the immediate vicinity of the TSS blocks gene transcription, while in the gene body, it may even stimulate transcription elongation [16]. In this regard, analysis of the frequency of the sign of probes in the genes of the immune group showed that the positive cluster phenomenon (i.e., the frequency of genes where all the probes were positive) was higher within gene body sequences, whereas the negative cluster phenomenon was higher in promoter sequences.

The most significant associations between variations in methylation and gene expression were observed in the upstream region from the TSS, which is not surprising since the CpG sites are frequently localized within gene promoters. However, significant associations were found in regions up to 500 bp downstream to the TSS. In fact, CGIs may be found inside the first and second exons, and they may control gene expression [16,29]. We have investigated methylation in the *SOCS1* and *SOCS3* genes, and they are examples of genes within CGIs that overrun the first and second exons. Several genes contain different ORFs, and methylation may influence the ORF chosen to start transcription. Yet, the analysis performed on the values associated with the variation in methyl scores and the frequency of negative probes returned results with differences in significance. The impact of the results is greater when the analysis is performed using the frequency of negative probes, indicating that unmethylation events play a major role in inflammatory cells during periodontal inflammation, as seen in Figures 3 and 4. The same tendency is observed when comparing the *p* values of the variation in methyl scores and the frequency of negative probes in genes with increased (mRNA > 0) and genes with decreased (mRNA < 0) differential expression in periodontitis and normal gingival tissues (Figure 6). The significance of the results is higher when the frequency of negative probes is tested as a parameter. The correlations between variations in gene expression and methylation should ideally be performed with expression data generated from the same individuals used to perform the methylation analysis. This would increase the likelihood to find correlations between variations in methylation and gene expression, as variations in methylation that cause subtle changes in gene expression could be detected. However, it is expected that in some specific genomic positions, variations in methylation will have a strong effect on gene expression. Variations in DNA methylation in those sites would consistently alter gene expression in humans. Therefore, it is plausible to infer that the probes that significantly correlated with gene expression represent the subset that have a significant effect on gene expression. Besides statistical significance, the fact that the number of probes significantly correlated with gene expression was larger in immune-inflammatory genes than in the other two groups and that these probes were located mainly near or upstream to the transcription start site corroborates with this assumption. This material may be a valuable source of information for other studies and in the validation of our results.

**Figure 8:**
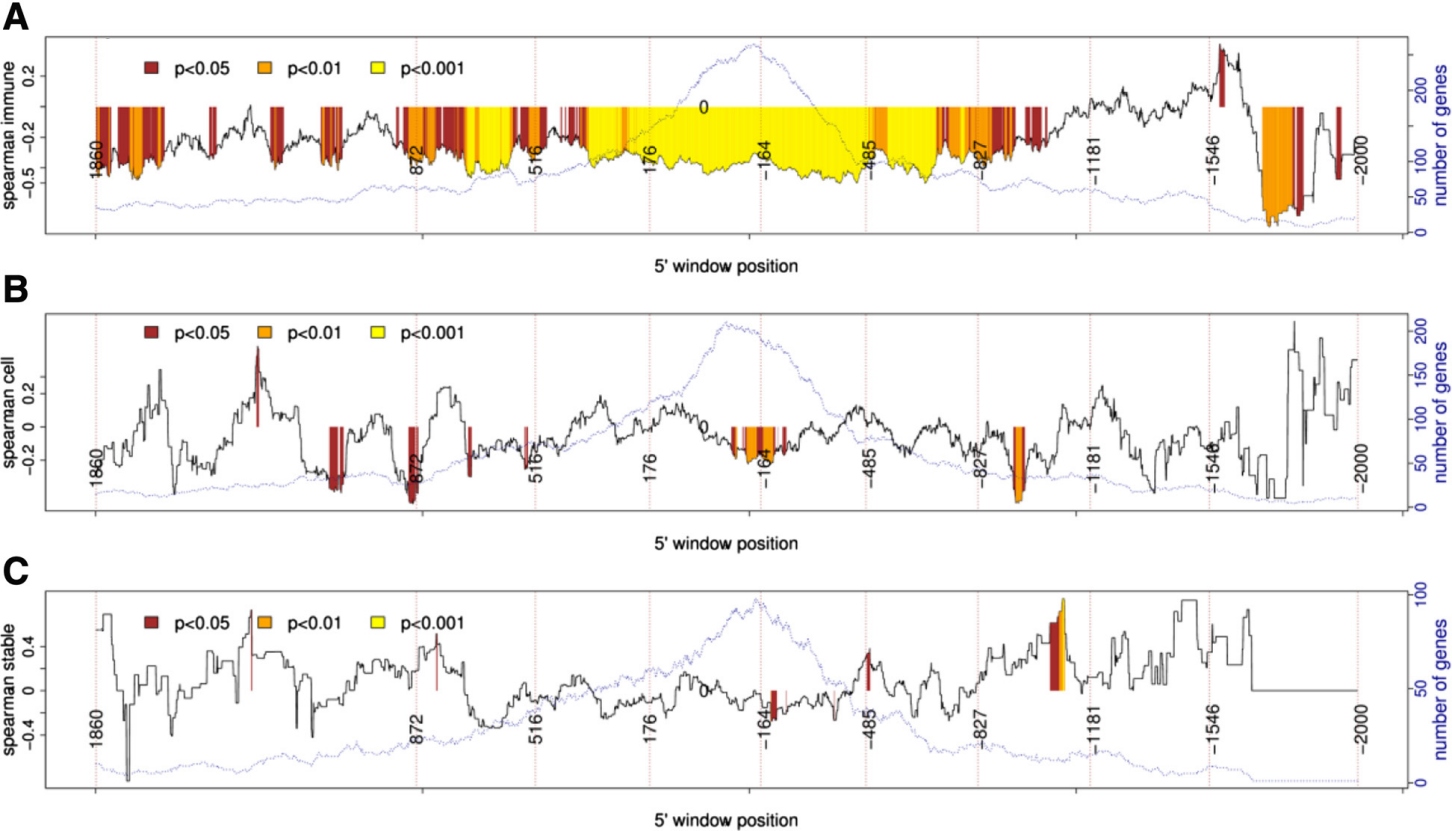
**Analysis of the correlation between variations in methylation (frequency of negative probes × methyl score) and mRNA.** The position of the probes in the immune **(A)**, stable **(B)**, and cell cycle **(C)** groups was matched using a sliding window of 140 bases (with a shift of one base) starting at a window with sequences ranging from +2,000 to +1,860 (within gene) towards 5′, ending at a window with a sequence range of −1,860 to −2,000 (promoter). The continuous black line shows the correlation values (Spearman's rank). Colors indicate the statistical significance of the correlation analysis. The dotted blue line indicates the number of genes in each window. Note that the largest areas with significant correlations (colored) are observed in the immune group.

The highest correlations between variations in methylation and gene expression were seen with a window size of 140 bp, which is close to nucleosome size. Although curious, it is hard to conclude whether the nucleosome presence (or absence) influenced this result. Indeed, in most cases, the substrate for DNA methyltransferase (DNMT3A/3B) is DNA associated to the nucleosome [30]. It is also worth commenting on the presence of somewhat irregular peaks and valleys in Figures 3A and 8A, evidenced by the horizontal dashed lines. The valleys appear in periods of approximately 350 bp, which are compatible with the size of the 140 bp window, passing from the beginning to the end of the nucleosome (i.e., 2 × nucleosome size = approximately 294) plus the linker DNA sequences, which can have up to 80 bp.

Finally, as well as being an important public health problem, chronic periodontitis is an interesting immune-inflammatory disease model due the landscapes of events that occur during its progression. Besides contributing to the understanding of the disease's pathogenesis, our results show that periodontitis can be a valuable model for understanding the basic mechanisms of the effects of DNA methylation on gene regulation and expression.

## Conclusions

Our results show that variations in DNA methylation between healthy and periodontitis cases are higher in genes related to the immune-inflammatory process. Also, the differences in DNA methylation variations are higher in sequences near the TSS, and variations in DNA methylation in immune-related genes correlate with variations in mRNA levels. Thus, DNA methylation must play a key role in periodontal inflammation, modulating chromatin regions that enable or not the mRNA transcription of immune-inflammatory genes related with periodontitis, what can be impacting the prognosis of disease.

## Methods

### Ethics statement

The study was performed in accordance with the current recommendations of the National Health Council - Ministry of Health of Brazil for research in human subjects and after approval from the Ethics Committee in Research of the School of Dentistry of Piracicaba - State University of Campinas (087/2013). Written informed consent was obtained from all volunteers.

### Collection of gingival tissue samples

Genomic DNA (gDNA) was purified from the gingival tissue of 12 chronic periodontitis and 11 healthy age- and gender-matched control individuals, with average age of 50.63 years and 50.42 years, in the groups respectively (Additional file 4: Table S2). All volunteers were recruited from clinics at the School of Dentistry of Piracicaba, University of Campinas (UNICAMP), and the following criteria were used to place individuals in either the chronic periodontitis group or the control group. (1) Chronic periodontitis group, fresh gingival biopsies were harvested from a single periodontal pocket affected by periodontitis in subjects undergoing periodontal surgery and who had more than three teeth demonstrating clinical attachment loss level (CAL) ≥ 5 mm and bleeding on probing [31]. (2) Control group, biopsies from clinically healthy gingival tissue were collected from non-inflamed sites of subjects who had surgery for a non-periodontal disease-related reason. These individuals exhibited no signs of periodontitis or gingival and/or periodontal inflammation, no bleeding on probing, and all teeth with a clinical attachment loss level ≤ 3.5 mm. The full mouth of individuals showed signs of chronic periodontal disease. The site chosen to be biopsied showed clinical signs of inflammation, CAL ≥ 5 mm in at least one of the six points around the tooth, and probing bleeding. The size of the specimens was approximately 2 × 2 mm and included junction epithelia and soft connective tissue. Excluded from both groups were individuals who smoked, individuals with systemic disorders that could affect the periodontal condition, those who had taken antibiotics and anti-inflammatory medication within the past 6 months, and women who were currently pregnant or lactating. The biopsies were collected and immediately stored on ice, then later frozen at −80°C.

### Genome-wide methylation chip

gDNA was isolated from cells of gingival tissue using the phenol sequence, and a total of 1 μg of gDNA was bisulfite converted by the EpiTect Bisulfite kit (Qiagen, Valencia, CA, USA) according to manufacturer's protocol. Following this, genome-wide methylation assays were performed on the Infinium®HumanMethylation 450 BeadChip from Illumina (Illumina, San Diego, CA, USA). This microarray chip covers 450,000 CpG sites per sample at single-nucleotide resolution, and each chip performed a total of 12 samples - one with 12 chronic periodontitis samples and the other with 11 control samples. The HM-450 K array employed different chemistry technologies (Infinium I and Infinium II) to guarantee depth of coverage of the methylation analysis. So, for each investigated CpG site, two site-specific probes were employed - one for the methylated locus, and another for the unmethylated locus (Infinium I) - and single-base extension of the hybridized probes incorporated a labeled ddNTP in order to quantify the levels of methylation and unmethylation in alleles (Infinium II). The microarray dataset is available in Gene Expression Omnibus [GSE53849]. (http://www.illumina.com/technology/infinium_methylation_assay.ilmn).

### Gene information and statistical analysis

The analysis of DNA methylation variation was performed in three different gene groups: immune = genes related to the immune-inflammatory system process (*n* = 1,284), cell cycle = genes related to the cell cycle process (*n* = 1,038); stable = a dataset of stably expressed genes (i.e., with consistent expression between physiological states and tissues [*n* = 575]) [32]. GO was used to obtain genes from the immune and cell cycle groups (The Gene Ontology Consortium, 2000, http://www.geneontology.org). Biological process terms were selected from the ‘Refine Selection’ menu of QuickGO (http://www.ebi.ac.uk/QuickGO) [33]. The gene characteristics (chromosome, genome position, and strand) were downloaded from Biomart (http://www.biomart.org) [34]. All probes encompassing the region from −2,500 to the end of the genes of the groups were selected for the initial analysis. For each probe, the methylation values of inflamed periodontitis gingival tissues (12 patients) and non-inflamed gingival tissues (11 patients) were compared using the Mann-Whitney-Wilcox test using the RinRuby R interpreter software [35]. *P* values of 59,999 probes were then filtered by the false discovery rate using the *q* value package of the R statistical package. Only probes with *q* values equal to or smaller than 0.05 and located in the sequences between +2,000 and −2,000 (immune = 5,422, cell cycle = 4,538, stable = 2,349) were used in further analysis. For each probe, we have subtracted the mean value of normal gingival tissue from periodontitis (i.e., chronic periodontitis − normal) to determine the sign (positive or negative) and the numeric value of the difference, here referred to as the methyl score. Therefore, probes with decreased methylation in periodontitis will be referred to as *negative probes* (negative methyl score), and probes with increased methylation will be referred as *positive probes* (positive methyl score).

### Methylation and gene expression

In order to investigate the association between the variations in methylation and mRNA levels in the three groups, we used the differential expression dataset between healthy and periodontitis gingival tissues from Demmer and collaborators [36]. The data was downloaded from the Gemma database (http://www.chibi.ubc.ca/Gemma/expressionExperiment/showExpressionExperiment.html?id=1585) [37]. The differential expression analysis was performed using the Affymetrix Gene Chip Human Genome U133 Plus 2.0 Array. Differential expression significance was performed by one-way analysis of variance (ANOVA) (*p* < 0.05) by examining gene expression signatures in healthy (unaffected sites) and diseased gingival tissues in 90 patients. In cases where there was more than one probe for the same gene, the mean values were used to obtain the variation in gene expression between normal and periodontitis cases. Genes with a differential expression of 0 (zero) were excluded from the analysis.

Genes were divided into two groups according to the sign of differential expression (positive, where mRNA levels increased in periodontitis and negative, where mRNA levels decreased in periodontitis). The differences in mean methyl scores and the frequency of negative probes between the positive and negative groups were compared using the Mann-Whitney-Wilcox test. Additionally, in order to fine map the areas with the highest correlation (Spearman correlation rank) between methylation and gene expression, the sequences spanning from 2,000 to −2,000 were scanned using a 140-bp sliding window (with a shift of one base).

## Competing interests

The authors declared that they have no competing interests.

## Authors' contributions

The experiments were conceived and designed by APS and SRPL. The experiments were performed by ACP and SRPL. Data were analyzed by APS, SRPL, and DDC. The paper was written by APS and SRPL. Biopsies were collected by ACP and MRM. All authors read and approved the final manuscript.

## Supplementary Material

Additional file 1: Figure S1 Methylation cluster phenomenon. The majority of probes within genes are positive. Click here for file

Additional file 2: Figure S2 Demethylation cluster phenomenon. The majority of probes within genes are negative. Click here for file

Additional file 3: Table S1 List of immune-related genes, cell cycle-related genes, and stable-expressed genes analyzed in this study. Click here for file

Additional file 4: Table S2 Demographic data of subjects. Click here for file
