# Supplementary figures and images for: High-throughput DNA analysis shows the importance of methylation in the control of immune inflammatory gene transcription in chronic periodontitis
Source: Clin Epigenetics. 2014 Aug 12;6(1):15. doi: 10.1186/1868-7083-6-15 (PMC4140141; doi:10.1186/1868-7083-6-15)

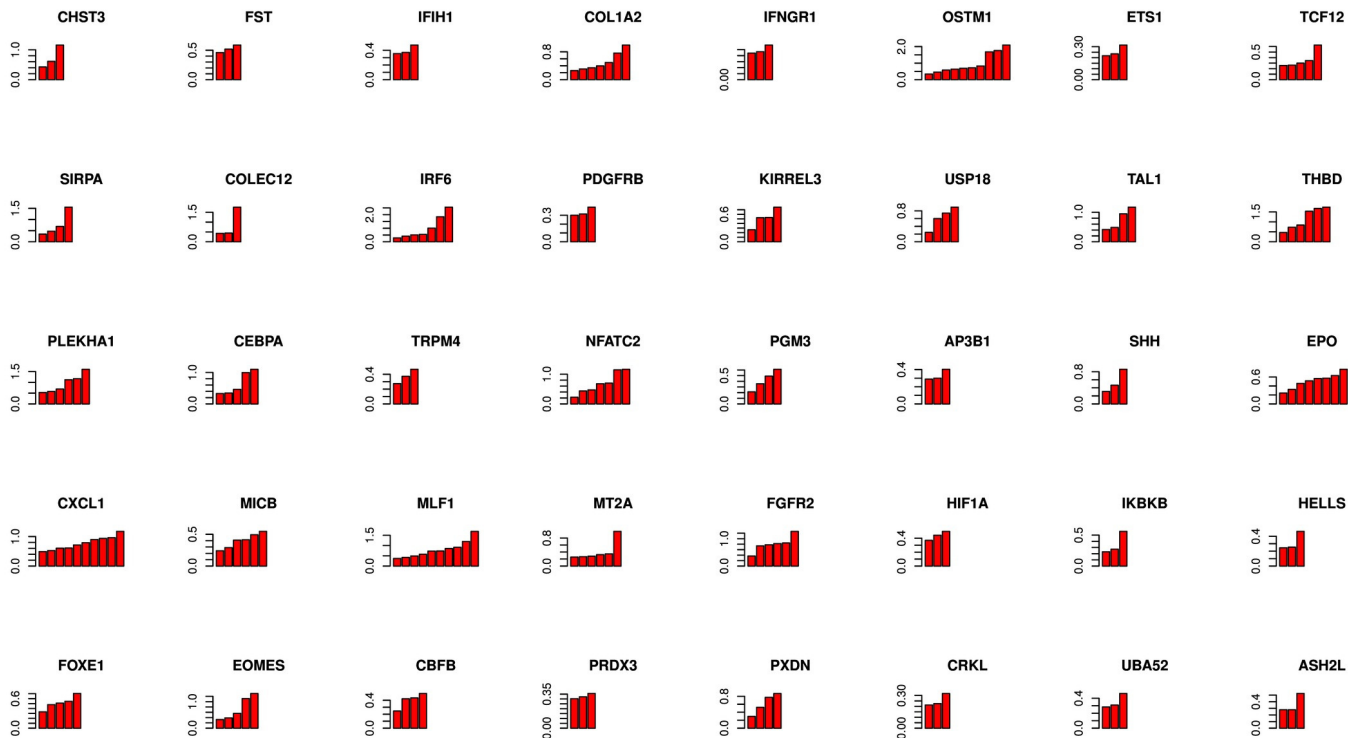

Supplement: Additional file 1: Figure S1 — Methylation cluster phenomenon. The majority of probes within genes are positive. [file 1868-7083-6-15-S1.pdf]

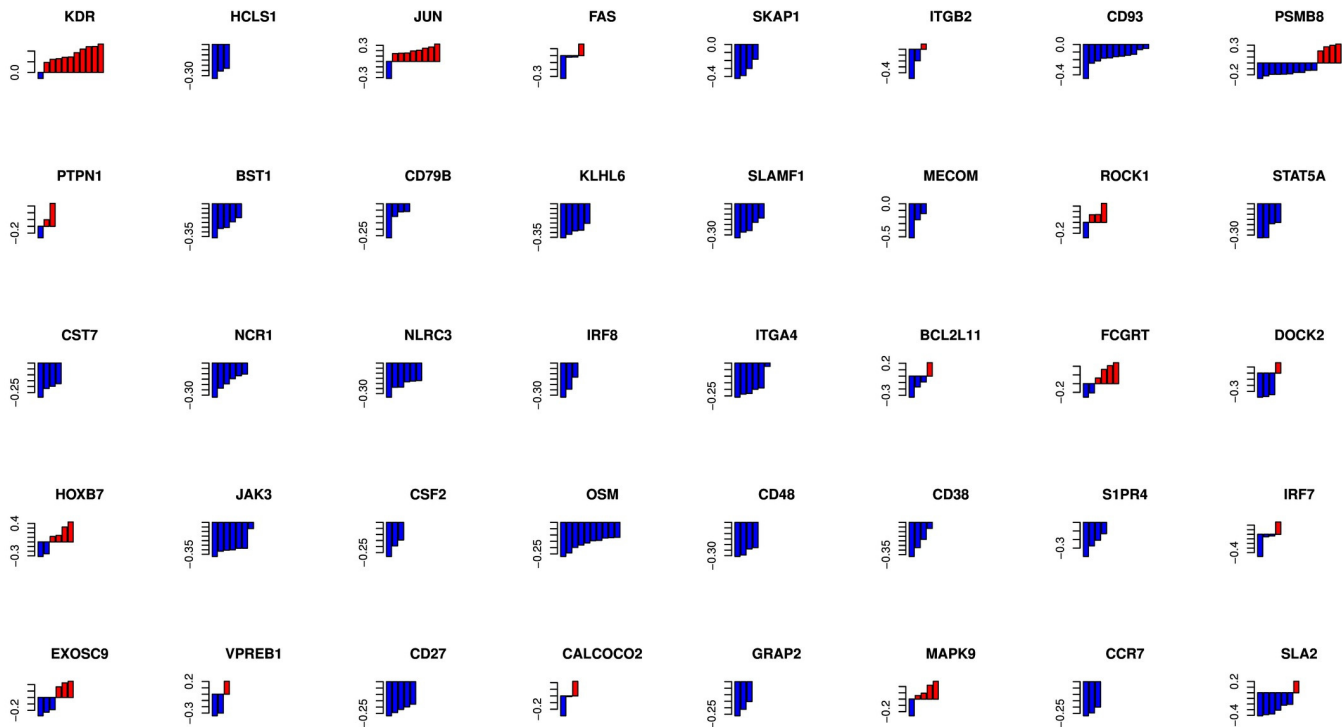

Supplement: Additional file 2: Figure S2 — Demethylation cluster phenomenon. The majority of probes within genes are negative. [file 1868-7083-6-15-S2.pdf]
