# Supplementary material for: High-throughput DNA analysis shows the importance of methylation in the control of immune inflammatory gene transcription in chronic periodontitis
Source: Clin Epigenetics. 2014 Aug 12;6(1):15. doi: 10.1186/1868-7083-6-15 (PMC4140141; doi:10.1186/1868-7083-6-15)
Supplement: Additional file 3: Table S1 — List of immune-related genes, cell cycle-related genes, and stable-expressed genes analyzed in this study. [file 1868-7083-6-15-S3.docx]

Additional file 3: Table S1

| **IMMUNE** | **CELLCYCLE** | **STABLE** |
| --- | --- | --- |
| AIM2 | A2M | AARS |
| AIMP1 | ACTB | ACIN1 |
| BCL2 | ADAMTS1 | ACP1 |
| BCL2L1 | ADAMTS13 | ACTA1 |
| BCL6 | ADAMTS8 | ACTA2 |
| BIRC2 | AIM2 | ACTB |
| BIRC3 | AIMP1 | ACTG1 |
| BMP2 | AKT1 | ACTR1A |
| C3 | ARAF | ACTR3 |
| C3AR1 | ATF2 | ADAR |
| C5 | B2M | AIMP1 |
| CARD18 | BCL2 | ALDH3A2 |
| CARD6 | BCL2L1 | ANP32B |
| CASP1 | BCL6 | AP1B1 |
| CASP5 | BIRC2 | APEX1 |
| CASP8 | BIRC3 | APOO |
| CCL1 | BMP2 | ARCN1 |
| CCL11 | BRAF | ARF1 |
| CCL13 | C3 | ARFGAP1 |
| CCL15 | C3AR1 | ARFGAP2 |
| CCL16 | C5 | ARHGAP1 |
| CCL17 | CARD18 | ARHGEF12 |
| CCL19 | CARD6 | ARL5A |
| CCL2 | CASP1 | ARL8B |
| CCL20 | CASP5 | ARPC2 |
| CCL21 | CASP8 | ARPC5 |
| CCL22 | CCL1 | ASMTL |
| CCL23 | CCL11 | ATF2 |
| CCL24 | CCL13 | ATF4 |
| CCL26 | CCL15 | ATG4B |
| CCL3 | CCL16 | ATMIN |
| CCL4 | CCL17 | ATP5B |
| CCL5 | CCL19 | ATP5E |
| CCL7 | CCL2 | ATP5H |
| CCL8 | CCL20 | ATP5J2 |
| CCR1 | CCL21 | ATP5J |
| CCR2 | CCL22 | ATP5L |
| CCR3 | CCL23 | ATP5O |
| CCR4 | CCL24 | ATRN |
| CCR5 | CCL26 | ATXN10 |
| CCR6 | CCL3 | AURKAIP1 |
| CCR7 | CCL4 | B2M |
| CCR8 | CCL5 | BBIP1 |
| CD14 | CCL7 | BCAS2 |
| CD40 | CCL8 | BECN1 |
| CD40LG | CCNA1 | BRD3 |
| CEBPB | CCNA2 | BSDC1 |
| CFLAR | CCNB1 | BTF3 |
| CHUK | CCNB2 | BUB3 |
| CIITA | CCND1 | C11ORF10 |
| CRP | CCND2 | C11ORF48 |
| CSF1 | CCND3 | C11ORF58 |
| CSF1R | CCNE1 | C14ORF2 |
| CSF2 | CCR1 | C16ORF61 |
| CSF3 | CCR2 | C19ORF53 |
| CTSB | CCR3 | C1ORF77 |
| CX3CL1 | CCR4 | C20ORF11 |
| CX3CR1 | CCR5 | C22ORF28 |
| CXCL1 | CCR6 | C2ORF28 |
| CXCL10 | CCR7 | CABIN1 |
| CXCL11 | CCR8 | CALM1 |
| CXCL12 | CD14 | CANX |
| CXCL13 | CD40 | CAP1 |
| CXCL2 | CD40LG | CAPNS1 |
| CXCL3 | CD44 | CAPZA1 |
| CXCL5 | CDC42 | CAPZB |
| CXCL6 | CDH1 | CBX1 |
| CXCL9 | CDK2 | CBX3 |
| CXCR1 | CDK4 | CCDC53 |
| CXCR2 | CDK6 | CCDC72 |
| CXCR4 | CDKN1A | CCND2 |
| F2 | CDKN1B | CCNI |
| FADD | CDKN1C | CCT8 |
| FAS | CDKN2A | CD63 |
| FASLG | CDKN2B | CD81 |
| FCER2 | CDKN2C | CDC5L |
| FCGR1A | CDKN2D | CDV3 |
| FOS | CEBPB | CFL1 |
| HSP90AA1 | CEBPD | CFLAR |
| HSP90AB1 | CFLAR | CHCHD2 |
| HSP90B1 | CHUK | CHD4 |
| IFNA2 | CIITA | CHMP2A |
| IFNB1 | CLEC3B | CIC |
| IFNG | CNTN1 | CIRBP |
| IKBKB | COL11A1 | CKM |
| IKBKG | COL12A1 | CLSTN1 |
| IL10 | COL14A1 | CNOT1 |
| IL10RA | COL15A1 | CNOT2 |
| IL10RB | COL16A1 | COPB1 |
| IL12A | COL1A1 | COPS6 |
| IL12B | COL4A2 | COPZ1 |
| IL13 | COL5A1 | COX4I1 |
| IL15 | COL6A1 | COX7A2L |
| IL16 | COL6A2 | COX7C |
| IL17A | COL7A1 | CRKL |
| IL17C | COL8A1 | CS |
| IL17F | CREB1 | CSNK1A1 |
| IL18 | CREBBP | CSNK2A1 |
| IL1A | CRK | CSNK2B |
| IL1B | CRP | CTCF |
| IL1R1 | CSF1 | CTDSPL |
| IL1RAP | CSF1R | CTNNA1 |
| IL1RN | CSF2 | CUX1 |
| IL20 | CSF3 | CYB5R3 |
| IL21 | CTGF | DAG1 |
| IL22 | CTNNA1 | DAZAP2 |
| IL23A | CTNNB1 | DCTN2 |
| IL23R | CTNND1 | DCTN6 |
| IL27 | CTNND2 | DDB1 |
| IL2RA | CTSB | DDX17 |
| IL2RG | CX3CL1 | DDX1 |
| IL3 | CX3CR1 | DDX27 |
| IL33 | CXCL1 | DDX50 |
| IL4 | CXCL10 | DEDD |
| IL4R | CXCL11 | DHX9 |
| IL5 | CXCL12 | DKC1 |
| IL5RA | CXCL13 | DRG1 |
| IL6 | CXCL2 | DUSP11 |
| IL6R | CXCL3 | DYNLRB1 |
| IL6ST | CXCL5 | EEF1A1 |
| IL7 | CXCL6 | EEF1B2 |
| IL8 | CXCL9 | EEF1D |
| IL9 | CXCR1 | EEF1G |
| IL9R | CXCR2 | EEF2 |
| IPAF | CXCR4 | EI24 |
| IRAK1 | DLK1 | EIF1 |
| IRF1 | E2F1 | EIF2B1 |
| IRF2 | ECM | EIF2S1 |
| IRF9 | ECM1 | EIF3A |
| ISG15 | EGFR | EIF3D |
| ITGB2 | EGR1 | EIF3F |
| KNG1 | ELK1 | EIF3G |
| LTA | EPOR | EIF3H |
| LTB | ETS1 | EIF4A2 |
| LY96 | ETS2 | EIF4B |
| MAP3K7 | F2 | EIF4G2 |
| MAPK1 | F2R | EIF4H |
| MAPK11 | FADD | ELAVL1 |
| MAPK12 | FAS | ENSA |
| MAPK13 | FASLG | ENY2 |
| MAPK3 | FCER2 | EPAS1 |
| MAPK8 | FCGR1A | ERH |
| MAPK9 | FN1 | ESD |
| MEFV | FOS | EWSR1 |
| MIF | GAPDH | FAM168B |
| MYD88 | GATA3 | FAM49B |
| NAIP | GHR | FAM53C |
| NAMPT | GRB2 | FAU |
| NFKB1 | GUSB | FBXL12 |
| NFKBIA | HAS1 | FKBP15 |
| NFKBIB | HMGA1 | FNTA |
| NLRC4 | HPRT1 | FRG1 |
| NLRC5 | HRAS | FTSJ3 |
| NLRP1 | HSP90AA1 | G3BP1 |
| NLRP12 | HSP90AB1 | GABARAPL2 |
| NLRP3 | HSP90B1 | GABARAP |
| NLRP4 | HSPA5 | GAPDH |
| NLRP5 | HSPB1 | GCN1L1 |
| NLRP6 | I | GDI1 |
| NLRP9 | ICAM1 | GGPS1 |
| NLRX1 | IFNA2 | GIPC1 |
| NOD1 | IFNAR1 | GLO1 |
| NOD2 | IFNB1 | GLRX5 |
| NOS2 | IFNG | GLT8D1 |
| NR3C1 | IFNGR1 | GNAS |
| OAS1 | II | GNB1 |
| OSM | IKBKB | GOSR1 |
| P2RX7 | IKBKG | GPX4 |
| PANX1 | IL10 | GRINA |
| PEA15 | IL10RA | GTF2F1 |
| PRLR | IL10RB | GTF2H5 |
| PSTPIP1 | IL12A | GTPBP6 |
| PTGS2 | IL12B | H2AFV |
| PTPN11 | IL13 | HADHA |
| PYCARD | IL15 | HARS |
| PYDC1 | IL16 | HAX1 |
| RAGE | IL17A | HDAC1 |
| RELA | IL17C | HDAC2 |
| RIPK2 | IL17F | HDAC3 |
| SELE | IL18 | HDGF |
| SPP1 | IL1A | HDLBP |
| STAT3 | IL1B | HERPUD1 |
| STUB1 | IL1R1 | HINT1 |
| SUGT1 | IL1RAP | HLA-A |
| TAB1 | IL1RN | HLA-C |
| TAB2 | IL20 | HMGB1 |
| TIRAP | IL21 | HMGN2 |
| TLR1 | IL22 | HMGN4 |
| TLR2 | IL23A | HNRNPA1 |
| TLR3 | IL23R | HNRNPA2B1 |
| TLR4 | IL27 | HNRNPAB |
| TLR5 | IL2RA | HNRNPD |
| TLR6 | IL2RG | HNRNPK |
| TLR7 | IL3 | HNRNPR |
| TLR9 | IL33 | HSD17B10 |
| TNF | IL4 | HSD17B12 |
| TNFRSF11B | IL4R | HSPA8 |
| TNFSF10 | IL5 | HUWE1 |
| TNFSF11 | IL5RA | IFITM3 |
| TNFSF13 | IL6 | IFNGR2 |
| TNFSF13B | IL6R | IFT20 |
| TNFSF14 | IL6ST | IK |
| TNFSF4 | IL7 | IKBKAP |
| TOLLIP | IL8 | ILF3 |
| TRAF6 | IL9 | IMMT |
| TXNIP | IL9R | IPO7 |
| VEGFA | INSR | IRF3 |
| XIAP | IPAF | ITGB1 |
| YY1 | IRAK1 | ITSN2 |
|  | IRF1 | JTB |
|  | IRF2 | KDM3B |
|  | IRF9 | KHDRBS1 |
|  | ISG15 | KIAA0174 |
|  | ITGA1 | KIAA0664 |
|  | ITGA2 | KLHL9 |
|  | ITGA3 | KRT10 |
|  | ITGA4 | LAMP1 |
|  | ITGA5 | LAPTM4A |
|  | ITGA6 | LIN37 |
|  | ITGA7 | LRRC40 |
|  | ITGA8 | LRRC47 |
|  | ITGAL | LSM14A |
|  | ITGAM | LSM3 |
|  | ITGAV | LSM6 |
|  | ITGB1 | MAGED2 |
|  | ITGB2 | MAML1 |
|  | ITGB3 | MAP1LC3B |
|  | ITGB4 | MAPKAPK2 |
|  | ITGB5 | MARK4 |
|  | JAK | MARS |
|  | JAK1 | MATR3 |
|  | JAK2 | MB |
|  | JAK3 | MBD1 |
|  | JNKK1 | MBD4 |
|  | JUN | MCL1 |
|  | JUNB | MCM3 |
|  | KAL1 | MDH2 |
|  | KNG1 | MED23 |
|  | KRAS | METTL9 |
|  | KSR1 | MGST2 |
|  | LAMA1 | MINK1 |
|  | LAMA2 | MKRN1 |
|  | LAMA3 | MLF2 |
|  | LAMB1 | MMADHC |
|  | LAMB3 | MOBKL1B |
|  | LAMC1 | MORF4L1 |
|  | LAMTOR3 | MORF4L2 |
|  | LDHA | MRPL16 |
|  | LRG1 | MRPL18 |
|  | LTA | MRPL3 |
|  | LTB | MRPL33 |
|  | LY96 | MRPL9 |
|  | MAP2K1 | MTCH1 |
|  | MAP2K2 | MTO1 |
|  | MAP2K3 | MXRA7 |
|  | MAP2K4 | MYBPC1 |
|  | MAP2K5 | MYH2 |
|  | MAP2K6 | MYL1 |
|  | MAP2K7 | MYL12A |
|  | MAP3K1 | MYL12B |
|  | MAP3K2 | MYL2 |
|  | MAP3K3 | MYL6 |
|  | MAP3K4 | NAA50 |
|  | MAP3K7 | NACA |
|  | MAP4K1 | NAP1L1 |
|  | MAPK | NARF |
|  | MAPK1 | NAT10 |
|  | MAPK10 | NBAS |
|  | MAPK11 | NBN |
|  | MAPK12 | NBR1 |
|  | MAPK13 | NCL |
|  | MAPK14 | NCOR1 |
|  | MAPK3 | NDUFB1 |
|  | MAPK6 | NDUFB8 |
|  | MAPK7 | NDUFC1 |
|  | MAPK8 | NDUFC2 |
|  | MAPK8IP2 | NEB |
|  | MAPK9 | NFE2L1 |
|  | MAPKAPK2 | NGFRAP1 |
|  | MAPKAPK3 | NGRN |
|  | MAX | NHP2L1 |
|  | MCL1 | NOL9 |
|  | MEF2C | NONO |
|  | MEFV | NPLOC4 |
|  | MEKK1 | NPM1 |
|  | MIF | NRD1 |
|  | MKNK1 | NSUN5 |
|  | MMP1 | OAZ1 |
|  | MMP10 | OS9 |
|  | MMP11 | OSBPL9 |
|  | MMP12 | OTUD4 |
|  | MMP13 | OXA1L |
|  | MMP14 | PABPC1 |
|  | MMP15 | PABPC3 |
|  | MMP16 | PAFAH1B1 |
|  | MMP2 | PARK7 |
|  | MMP3 | PCBP2 |
|  | MMP7 | PCGF3 |
|  | MMP8 | PCMT1 |
|  | MMP9 | PCNP |
|  | MOS | PFDN1 |
|  | MPL | PFDN2 |
|  | MST1 | PGAM1 |
|  | MYC | PGK1 |
|  | MYD88 | PHB2 |
|  | NAIP | PHF3 |
|  | NAMPT | PI4KB |
|  | NCAM1 | PLA2G16 |
|  | NFATC4 | PLEKHM1 |
|  | NFKB1 | PNN |
|  | NFKBIA | PNPO |
|  | NFKBIB | PNRC1 |
|  | NLRC4 | PNRC2 |
|  | NLRC5 | POLDIP3 |
|  | NLRP1 | POLR2B |
|  | NLRP12 | POLR2C |
|  | NLRP3 | POLR2F |
|  | NLRP4 | POLR2G |
|  | NLRP5 | POMP |
|  | NLRP6 | PPCS |
|  | NLRP9 | PPIA |
|  | NLRX1 | PPM1G |
|  | NOD1 | PPP1R8 |
|  | NOD2 | PPP2R5A |
|  | NONO | PPP3CB |
|  | NOS2 | PPP6C |
|  | NR3C1 | PPP6R3 |
|  | NRAS | PQLC1 |
|  | OAS1 | PRDM4 |
|  | OSM | PRKAR2A |
|  | P2RX7 | PSMA1 |
|  | PAK1 | PSMA3 |
|  | PANX1 | PSMA6 |
|  | PDGFRA | PSMA7 |
|  | PEA15 | PSMB1 |
|  | PECAM1 | PSMB4 |
|  | PGK1 | PSMC5 |
|  | PIAS1 | PSMD13 |
|  | PIAS2 | PSMD2 |
|  | PPIH | PSMD4 |
|  | PRDX6 | PSMF1 |
|  | PRL | PTK2 |
|  | PRLR | PTMA |
|  | PSTPIP1 | PUM1 |
|  | PTGS2 | PWP1 |
|  | PTPN1 | QKI |
|  | PTPN11 | QRICH1 |
|  | PTPRC | RAB11A |
|  | PYCARD | RAB1A |
|  | PYDC1 | RAB2A |
|  | RAC1 | RAB3GAP2 |
|  | RAF1 | RAB5A |
|  | RAGE | RAB6A |
|  | RB1 | RAC1 |
|  | RELA | RAD17 |
|  | RIPK2 | RAD23A |
|  | RNA | RAF1 |
|  | RPLP0 | RALB |
|  | SELE | RBBP7 |
|  | SELL | RBM22 |
|  | SELP | RBMX |
|  | SFN | RELA |
|  | SGCE | RER1 |
|  | SH2B1 | RGL2 |
|  | SH3 | RHEB |
|  | SMAD1 | RHOA |
|  | SMAD2 | RHOT1 |
|  | SMAD3 | RING1 |
|  | SMAD4 | RNF10 |
|  | SMAD5 | RNF220 |
|  | SOCS1 | RNPS1 |
|  | SOCS2 | RPL10 |
|  | SOCS3 | RPL10A |
|  | SOCS4 | RPL11 |
|  | SOCS5 | RPL12 |
|  | SP1 | RPL13 |
|  | SPARC | RPL13A |
|  | SPG7 | RPL14 |
|  | SPI1 | RPL15 |
|  | SPP1 | RPL17 |
|  | SRC | RPL18 |
|  | STAM | RPL19 |
|  | STAT | RPL21 |
|  | STAT1 | RPL22 |
|  | STAT2 | RPL23 |
|  | STAT3 | RPL23A |
|  | STAT4 | RPL24 |
|  | STAT5 | RPL26 |
|  | STAT5A | RPL27 |
|  | STAT5B | RPL27A |
|  | STAT6 | RPL28 |
|  | STUB1 | RPL29 |
|  | SUGT1 | RPL3 |
|  | TAB1 | RPL30 |
|  | TAB2 | RPL31 |
|  | TFRC | RPL32 |
|  | TGFBI | RPL34 |
|  | THBS1 | RPL35 |
|  | THBS2 | RPL36A |
|  | THBS3 | RPL37A |
|  | TIMP1 | RPL37 |
|  | TIMP2 | RPL38 |
|  | TIMP3 | RPL39 |
|  | TIRAP | RPL4 |
|  | TLR1 | RPL41 |
|  | TLR2 | RPL5 |
|  | TLR3 | RPL6 |
|  | TLR4 | RPL7 |
|  | TLR5 | RPL7A |
|  | TLR6 | RPL9 |
|  | TLR7 | RPLP0 |
|  | TLR9 | RPLP1 |
|  | TNC | RPLP2 |
|  | TNF | RPP30 |
|  | TNFRSF11B | RPS10 |
|  | TNFSF10 | RPS11 |
|  | TNFSF11 | RPS12 |
|  | TNFSF13 | RPS13 |
|  | TNFSF13B | RPS14 |
|  | TNFSF14 | RPS15A |
|  | TNFSF4 | RPS15 |
|  | TOLLIP | RPS16 |
|  | TP53 | RPS17 |
|  | TRAF6 | RPS18 |
|  | TXNIP | RPS19 |
|  | TYK2 | RPS2 |
|  | USF1 | RPS20 |
|  | VCAM1 | RPS23 |
|  | VCAN | RPS24 |
|  | VEGFA | RPS25 |
|  | VTN | RPS27 |
|  | XIAP | RPS27A |
|  | YY1 | RPS28 |
|  |  | RPS29 |
|  |  | RPS3 |
|  |  | RPS3A |
|  |  | RPS4X |
|  |  | RPS5 |
|  |  | RPS6 |
|  |  | RPS7 |
|  |  | RPS8 |
|  |  | RPS9 |
|  |  | RPSA |
|  |  | RRAGA |
|  |  | RRN3 |
|  |  | RRP1B |
|  |  | RTF1 |
|  |  | RTN4 |
|  |  | RUFY1 |
|  |  | RXRA |
|  |  | SAFB |
|  |  | SCFD1 |
|  |  | SEC11A |
|  |  | SEC13 |
|  |  | SEC31A |
|  |  | SEL1L |
|  |  | SERBP1 |
|  |  | SERF2 |
|  |  | SERP1 |
|  |  | SET |
|  |  | SETD1B |
|  |  | SF1 |
|  |  | SF3A1 |
|  |  | SF3B3 |
|  |  | SKP1 |
|  |  | SLC12A7 |
|  |  | SLC25A3 |
|  |  | SLC25A6 |
|  |  | SLN |
|  |  | SMARCE1 |
|  |  | SMC1A |
|  |  | SMPX |
|  |  | SNRNP200 |
|  |  | SNRNP27 |
|  |  | SNRPB2 |
|  |  | SNRPD2 |
|  |  | SNW1 |
|  |  | SNX3 |
|  |  | SON |
|  |  | SPAG7 |
|  |  | SPARCL1 |
|  |  | SPSB3 |
|  |  | SQSTM1 |
|  |  | SRP14 |
|  |  | SRP9 |
|  |  | SRRM1 |
|  |  | SSBP1 |
|  |  | SSR2 |
|  |  | ST13 |
|  |  | STARD7 |
|  |  | STAU1 |
|  |  | STX16 |
|  |  | SUMO1 |
|  |  | SUMO2 |
|  |  | SUPT4H1 |
|  |  | SUPT5H |
|  |  | SUPT6H |
|  |  | TBC1D9B |
|  |  | TBCB |
|  |  | TBCC |
|  |  | TCEA1 |
|  |  | TCEB3 |
|  |  | TERF2IP |
|  |  | TEX261 |
|  |  | TFG |
|  |  | THAP11 |
|  |  | THAP7 |
|  |  | THYN1 |
|  |  | TM9SF4 |
|  |  | TMEM222 |
|  |  | TMEM66 |
|  |  | TMEM93 |
|  |  | TMUB2 |
|  |  | TNNC2 |
|  |  | TNPO3 |
|  |  | TOMM34 |
|  |  | TOX4 |
|  |  | TPD52L2 |
|  |  | TPI1 |
|  |  | TPM2 |
|  |  | TPT1 |
|  |  | TRAPPC4 |
|  |  | TRIM27 |
|  |  | TRIM8 |
|  |  | TRIOBP |
|  |  | TRPC4AP |
|  |  | TSN |
|  |  | TUBA1B |
|  |  | TXNL1 |
|  |  | U2AF1 |
|  |  | UBA2 |
|  |  | UBA52 |
|  |  | UBAP2L |
|  |  | UBB |
|  |  | UBC |
|  |  | UBE2B |
|  |  | UBE2D2 |
|  |  | UBE2L3 |
|  |  | UBE2N |
|  |  | UBE3A |
|  |  | UBL3 |
|  |  | UBN1 |
|  |  | UBXN6 |
|  |  | UFC1 |
|  |  | UFM1 |
|  |  | UQCRB |
|  |  | USP24 |
|  |  | USP34 |
|  |  | USP4 |
|  |  | USP7 |
|  |  | USP9X |
|  |  | UXT |
|  |  | VAPB |
|  |  | VASP |
|  |  | VCP |
|  |  | VDAC2 |
|  |  | WDR73 |
|  |  | WDR77 |
|  |  | WDR82 |
|  |  | XPNPEP1 |
|  |  | XRCC6 |
|  |  | YWHAQ |
|  |  | YY1 |
|  |  | YY1AP1 |
|  |  | ZC3H14 |
|  |  | ZCCHC8 |
|  |  | ZFP64 |
|  |  | ZMIZ2 |
|  |  | ZMYM4 |
|  |  | ZNF207 |
|  |  | ZNF212 |
|  |  | ZNF32 |
|  |  | ZNF544 |
|  |  | ZNF706 |
|  |  | ZYX |
