# Supplementary material for: High-throughput DNA analysis shows the importance of methylation in the control of immune inflammatory gene transcription in chronic periodontitis
Source: Clin Epigenetics. 2014 Aug 12;6(1):15. doi: 10.1186/1868-7083-6-15 (PMC4140141; doi:10.1186/1868-7083-6-15)
Supplement: Additional file 4: Table S2 — Demographic data of subjects. [file 1868-7083-6-15-S4.docx]

Additional file 4: Table S2 Demographic data of subjects

|  | **Periodontitis** | **Control** |
| --- | --- | --- |
| **Male** | 05 | 05 |
| **Female** | 07 | 06 |
| **mean age** | 50,63 | 50,42 |
| **SD age** | 7,89 | 8,35 |
